# Revealing the Complexity of Host-Parasite Relationships Between Syringophilid Mites and Sunbirds in Their Global Range

**DOI:** 10.3390/ani15010110

**Published:** 2025-01-06

**Authors:** Bozena Sikora, Markus Unsoeld, Roland R. Melzer, Stefan Friedrich, Martin Hromada

**Affiliations:** 1 Department of Animal Morphology, Faculty of Biology, Adam Mickiewicz University, 61-614 Poznan, Poland; 2 Sektion Ornithology, SNSB-Bavarian State Collection of Zoology, 81247 Munich, Germany; unsoeld@snsb.de; 3 Sektion Arthropoda Varia, SNSB-Bavarian State Collection of Zoology, 81247 Munich, Germany; melzer@snsb.de (R.R.M.); friedrich@snsb.de (S.F.); 4 Faculty of Biology, Ludwig Maximilian University of Munich, 82152 Planegg-Martinsried, Germany; 5 GeoBio-Center, Ludwig Maximilian University of Munich, 80333 Munich, Germany; 6 Laboratory and Museum of Evolutionary Ecology, Department of Ecology, Faculty of Humanities and Natural Sciences, University of Prešov, 080 01 Prešov, Slovakia; hromada.martin@gmail.com

**Keywords:** Acari, Aves, biodiversity, birds, ectoparasites, Nectariniidae, Syringophilidae

## Abstract

This article emphasises the importance of parasitological research in understanding ecological dynamics and biodiversity conservation through a global analysis of quill mites (Syringophilidae) parasitising Sunbirds (Nectariniidae). Data from 764 Sunbird individuals across seventy-six species revealed twelve quill mite species, including three newly described species: *Aulonastus aethopygus* Sikora and Unsoeld sp. n., *Syringophiloidus haeckeli* Sikora and Unsoeld sp. n., and *Aulonastus arachnotherus* Sikora and Unsoeld sp. n. A bipartite network analysis indicated a low connectance of 0.10, suggesting that only 10% of potential bird–parasite connections are realised. This high specialisation is further supported by an H_2′_ index of 0.94 and a C score of 0.83, indicating low co-occurrence among mite species. The temperature of nestedness at 13.49 suggests a well-organised network structure. Additionally, normalised specialisation (d’) ranged from 0.60 to 1, reflecting unique host–parasite interactions. High modularity (likelihood = 0.80) with nine modules was identified, with hosts ranging from one to seven. The study concludes by discussing the host–parasite dynamics and their ecological implications within this system.

## Simple Summary

This study explores the ecological relationships between quill mites (Syringophilidae) and Sunbirds (Nectariniidae), revealing an example of host–parasite co-evolution. Through the analysis of 764 Sunbirds across seventy-six species, researchers identified twelve quill mite species, including three new ones for science. Our findings highlight the high specialisation of these mites to their avian hosts which is reflected in unique host–parasite relationships, low co-occurrence among mite species, and a well-organised interaction network. The research also uncovers distinct ecological patterns, such as the habitat specificity of mites on different feather types and the evolutionary dispersal of mites alongside Sunbirds.

## 1. Introduction

The passerine family Nectariniidae comprises 136–148 species in 16 genera, including Sunbirds and Spiderhunters (Passeriformes: Nectariniidae) [1,2,3]. These avian species are typically small- to medium-sized and are distinguished by their slender, predominantly downward-curving bills. While Spiderhunters generally display an olive-yellow colouration with streaks on their lower bodies, many male Sunbird species are noted for their exceptionally vibrant and iridescent plumage [4]. Their geographic distribution extends from sub-Saharan Africa (including Egypt) to the Middle East, South Asia, Southeast Asia, southern China, Indonesia, New Guinea, and northern Australia, with the greatest species diversity found in equatorial regions [2]. Sunbirds thrive in a wide range of habitats, from arid savannas to lush tropical rainforests, and are found at elevations from sea level to 4900 m [5]. They are predominantly monogamous and exhibit biparental care, with some species even demonstrating cooperative breeding behaviour, where conspecifics assist in rearing young. The Nectariniidae family is a crucial component of the passeroid radiation within oscine passerines and is likely the sister group to the flowerpeckers (Dicaeidae), representing some of the most ancient lineages within the Passeroidea [4,6].

Quill mites of the family Syringophilidae Lavoipierre (Acariformes: Prostigmata: Cheyletoidea) are obligate parasites of birds [7]. All species, without exception, inhabit the quills of feathers where they develop and mate. They feed on the fluid tissue surrounding the quill, piercing its wall with their long, needle-like chelicerae [8,9,10]. The dispersing individuals are mature and fertilised females, which most commonly colonise new host individuals through vertical transfer [11,12,13]. Quill mites exhibit high specificity in relation to their hosts, where the vast majority of Syringophilidae species are mono- or oligoxenous, and regarding the habitats they colonise [14]. The quill mite fauna of the family Syringophilidae, associated with Sunbirds, has been the subject of study over the last decade. Currently, the syringophilid fauna associated with Sunbirds and Spiderhunters includes nine species. Most of these species have been recorded primarily from Sub-Saharan hosts, with only three species reported from non-African nectariniids [15,16,17,18,19].

This paper presents a comprehensive revision of the quill mite fauna associated with Sunbirds. We describe three new species and document several new host records for previously described mites. Additionally, we provide an identification key for all syringophilid species associated with Nectariniidae. Furthermore, we analyse the host–parasite relationships within the system comprising syringophilid mites and Sunbirds. Our approach is related to network analysis, which offers a powerful tool for unravelling the intricate relationships between Sunbirds and their quill mite parasites. By constructing and examining bipartite networks, we gain deeper insights into the specialisation and co-evolution of these host–parasite pairs. For instance, metrics such as the specialisation index (H_2′_) and modularity allow us to identify distinct clusters of host–parasite interactions, which reveal how certain Sunbird species may have evolved specific defences or susceptibilities to particular quill mite species [20,21]. Additionally, evaluating the nestedness of these networks provides a further understanding of the hierarchical organisation of parasite–host interactions, where some mites may be generalists while others are highly specialised [22,23,24]. This approach is crucial for understanding the ecological dynamics within Sunbird communities and has significant implications for biodiversity conservation.

## 2. Materials and Methods

### 2.1. Mites Collection, Preparation, Description, and Deposition

**Figure 1:**
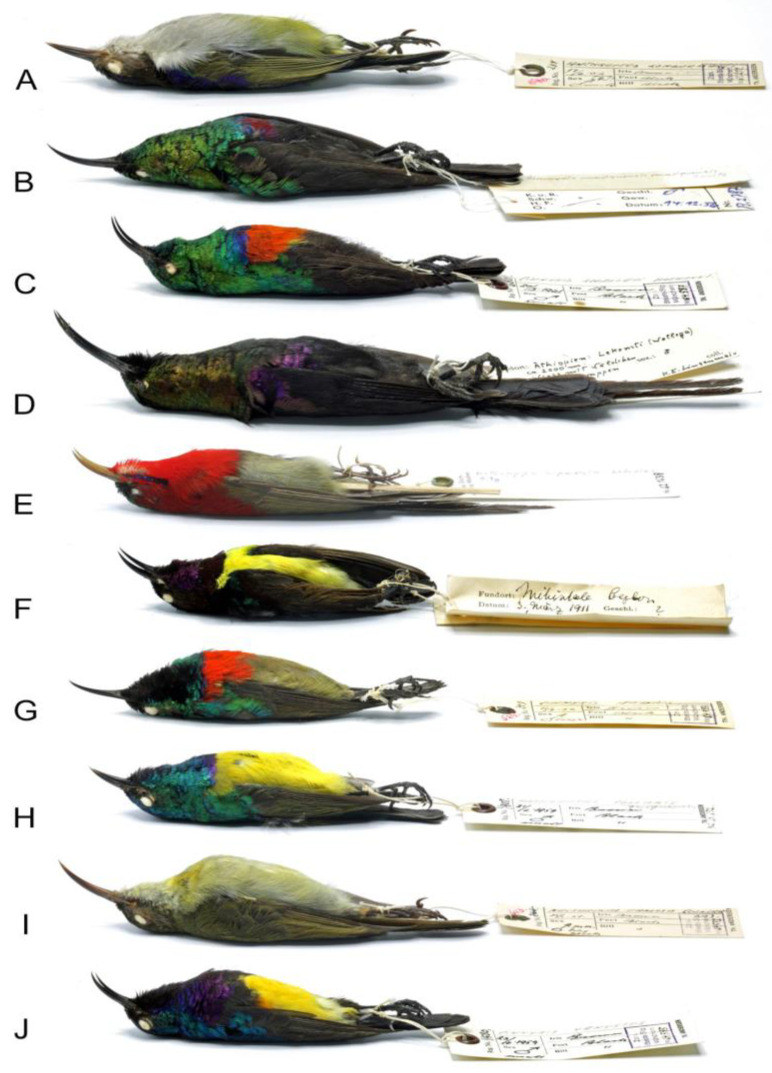
Examples of bird species deposited in SNSB-ZSM and infested by quill mites. (**A**) *Anthreptes longuemarei* (Lesson); (**B**) *Cinnyris mariquensis* Smith; (**C**) *Cinnyris shelleyi* Alexander; (**D**) *Nectarinia tacazze* (Stanley); (**E**) *Aethopyga siparaja* (Raffles); (**F**) *Leptocoma zeylonica* (Linnaeus); (**G**) *Cinnyris mediocris* Shelley; (**H**) *Hedydipna collaris* (Vieillot); (**I**) *Cyanomitra olivacea* (Smith); (**J**) *Cinnyris venustus* (Shaw).

New mite material used in the current study has been collected from dry bird skins housed in the Bavarian State Collection of Zoology, Munich, Germany (SNSB-ZSM) (Figure 1). From each bird specimen, about ten contour feathers (near the cloaca region), two under-tail coverts, two upper-tail coverts, and one secondary covert were examined under a stereomicroscope. Before mounting, mites were softened in Nesbitt’s solution at room temperature for three or four days [14]. The mites were then mounted on slides in Hoyer’s medium [25]. Slide-mounted mites were examined under a ZEISS Axioscope (Carl-Zeiss AG, Oberkochen, Germany) light microscope equipped with differential interference contrast (DIC) optics and camera lucida.

In the descriptions, the idiosomal setation follows Grandjean [26] as adapted for Prostigmata by Kethley [27]. The nomenclature of leg chaetotaxy follows that proposed by Grandjean [28]. The morphological terminology follows Kethley [1] and Skoracki [14]. All measurements are given in micrometres. Measurements (ranges) of paratypes are given in brackets following data for a holotype.

All mite material used in the present study is deposited in the two institutions: AMU—Adam Mickiewicz University, Poznan, Department of Animal Morphology and ZSM-SNSB—Bavarian State Collection of Zoology, Munich, Germany.

### 2.2. Statistical Analysis

Descriptive statistics were computed using Quantitative Parasitology v.3.0 on the Web [29,30,31]. To describe patterns within the studied host–parasite ecological two-way web, the ’bipartite’ package for R (ver. 4.3.1.) was used [32]. This approach quantifies the ecological connections between parasites and their hosts [21]. In our matrix, parasite prevalence was utilised as a quantitative index. In the first step, we calculate connectance (connection ratio) in a bipartite network. This measure determines the ratio of the number of actual connections in the network to the maximum possible number of connections. This is an indicator of network density and is expressed as a value between 0 and 1, where 1 means that every possible element from one part of the network (parasites) is connected to every element from the other part of the network (host) and 0 means that there are no connections. Next, we calculate C score, i.e., a measure describing the tendency towards the non-co-occurrence of pairs of species. A high value of this index indicates a tendency for species to not co-occur, which may suggest competition or other forms of mutual avoidance, while a low value of C score indicates that species tend to co-occur more often than would be expected based on random distribution. To determine whether parasites are generalists or specialists, we will use the H_2′_ measure. This is a statistic ranging from 0 to 1, where a value of 0 means that all species are generalists (i.e., they interact with many other hosts) and a value of 1 means that all species are specialists (i.e., each species only interacts with one host). The null.t.test was employed to check whether the observed H_2_′ values significantly deviated from random values [32]. We also calculated nestedness. It is a measure of order in the network. If parasites with a low number of interactions with hosts share these interactions with other hosts with a larger number of interactions, the network is considered nested. Nestedness temperature is a measure of how much the actual nestedness of a network differs from a perfectly nested network. For a perfectly nested network, the temperature would be 0. A higher temperature means that the nesting is less than perfect. As a measure of the functional diversity of parasites, we used d’. The d’ is a measure normalised to the maximum possible diversity for a given species in the network. This value ranges from 0 to 1, with 1 indicating maximum functional diversity. If d’ for a species = 1, it means that it has maximum functional diversity, which means that its way of interacting with other species is very diverse and unique compared to other species in the network. Finally, we also calculated modularity as a probability value that measures how well the modular structure fits the data. A value close to 1 indicates that the modular model has a relatively good fit to the observed data, suggesting that modularity is a significant feature of this network.

### 2.3. Bird Systematics, Host Specificity, and Zoogeographical Regions

The scientific names and systematics of the birds follow Clements et al. [33] and Winkler et al. [2]. The distribution of the host species follows BirdLife International [34]. Host specificity follows Ciara et al. [35]. Zoogeographical regions follow Holt et al. [36] and Ficetola et al. [37].

## 3. Results

### 3.1. Revision of Quill Mite Species Associated with Sunbirds

*Aulobia afroanthreptes* Skoracki and Zmudzinski, 2018

Hosts and Distribution: Uluguru Violet-backed Sunbird *Anthreptes neglectus* Neumann and Western Violet-backed Sunbird *Anthreptes longuemarei* (Lesson), both from Tanzania [18].

*Aulobia anthreptes* Skoracki and Glowska, 2008

Hosts and distribution: Plain-throated Sunbird *Anthreptes malacensis* (Scopoli) from unprecise locality in SE Asia [15] and Indonesia (Java) (current study).

New Material Examined

Ex quill of wing covert of *Anthreptes malacensis* (host reg. no. ZSM 27.739/V216, male); Indonesia: Java, West Java Province, Cheribon, 9 May 1927, coll. Menden—six females deposited in the AMU and three females in the ZSM.

*Aulobia nectariniae* Skoracki and Glowska, 2008

Hosts and distribution: Olive-backed Sunbird *Cinnyris jugularis* (Linnaeus) from Papua New Guinea ([15], current study), Mariqua Sunbird *Cinnyris mariquensis* Smith and Shelley’s Sunbird *Cinnyris shelleyi* Alexander, both from Tanzania [18], Palestine Sunbird *Cinnyris osea* Bonaparte (new host) from Palestine (current study), Black Sunbird *Leptocoma aspasia* (Lesson and Garnot) (=*L. sericea* (Lesson)) from Papua New Guinea ([15], current study).

New Material Examined

Ex quill of wing covert of *Cinnyris osea* (host reg. no. ZSM 17.1276/V194, male); Palestine: Jericho, 2 April 1909, coll. Laubmann—six females and one male deposited in the AMU, two females and one male in the ZSM.

Ex quill of wing covert of *Cinnyris jugularis* (host reg. no. ZSM uncatalogued); Papua New Guinea: Madang Province, Astrolabe Bay, coll. C.B. Hagen—one female deposited in the AMU. Ex same habitat and host species (host reg. no. ZSM 11.845/V178-180, female); Papua New Guinea: August 1910, coll. L. von Wiedenfeld—six females deposited in the AMU.

Ex quill of wing covert of *Leptocoma aspasia* (host reg. no. ZSM 14.719/V183, male); Indonesia: Misol Island, August 1911, coll. O. Tauern—five females and one male deposited in the AMU, two females in the ZSM. Ex same habitat and host species (host reg. no. ZSM 14.719/V184), and locality, August 1911, coll. O. Tauern—three females and two males deposited in the AMU. Ex same habitat and host species (host reg. no. ZSM 11.850/V187); Papua New Guinea: September 1910, coll. L. von Wiedenfeld—three females and one male deposited in the AMU, three females in the ZSM. Ex same habitat and host species (host reg. no. ZSM uncatalogued, female ad.); Papua New Guinea: Madang Province, Astrolabe-Bay, coll. C.B. Hagen—three females deposited in the AMU.

*Aulonastus nectariniiphilus* Skoracki and Zmudzinski, 2018

Hosts and distribution: Plain-backed Sunbird *Anthreptes reichenowi* Gunning from Tanzania and Tacazze Sunbird *Nectarinia tacazze* (Stanley) from Ethiopia [18].

*Aulonastus aethopygus* Sikora and Unsoeld sp. n.

(Figure 2)

Description. Female, holotype. Total body length 360 (430 in one paratype). Gnathostoma. Infracapitulum apunctate. Each medial branch of peritremes with two chambers, and each lateral branch with five or six chambers. Stylophore apunctate, 90 (80) long. Idiosoma. Propodonotal shield rectangular in shape, weakly sclerotised, apunctate, bearing bases of setae *ve*, *si*, *se*, and *c1.* Setae *ve* and *si* subequal in length. Setae *c1* not significantly (1.1 times) longer than *se*, both setae situated at same transverse level. Length ratio of setae *d2:c1* 1:1.3–1.4. Hysteronotal shield absent. Pygidial shield apunctate. Setae *f2* 1.5–1.6 times longer than *f1.* Setae *h2* 5.3–6.6 times longer than *f2.* Length ratio of setae *ag1*:*ag2*:*ag3* 1:1.8–2.8:2.3–3.5 Genital plate absent. Both pairs of genital setae subequal in length. All coxal fields apunctate. Setae *3c* about 2–2.5 times longer than *3b.* Legs. Fan-like setae *p’* and *p”* of legs III and IV with five or six tines. Lengths of setae: *ve* 15 (15), *si* 15 (20), *se* 135 (135), *c1* 150 (145), *c2* 130 (125), *d1* 15 (20), *d2* 115 (105), *e2* 25 (25), *f1* 15 (15), *f2* 30 (35), *h1* 15 (15), *h2* 210, *ps1* 15 (15), *g1* and *g2* 15 (20), *ag1* 30 (35), *ag2* (35), *ag3* 70 (65), *3b* 10 (15), *3c* 25 (30), *l’RIII–IV* 10 (10).

Male. Not found.

Type Material

Female holotype and one female paratype from under-tail covert quill of Crimson Sunbird *Aethopyga siparaja* (Raffles) (Passeriformes: Nectariniidae) (host reg. no. ZSM–30.243/V168); Philippines: Insel Luzon, Manila, coll. I. Marschal.

Type Material Deposition

Female holotype is deposited in the AMU, and female paratype is in the ZSM-SNSB.

Etymology

The name *aethopygus* is derived from the generic name of the host and is formed as an adjective agreeing in gender and case with the masculine genus name *Aulonastus*. It reflects the association of this species with hosts from the genus *Aethopyga*.

Differential Diagnosis

*Aulonastus aethopygus* sp. n. is morphologically similar to *A. lusciniae* Skoracki, 2002 described from *Luscinia luscinia* (Passeriformes: Turdidae) from Slovakia [38]. In females of both species, setae *ve* and *si* are subequal in length; setae *c1* and *se* are subequal in length, and setae *h2* are distinctly longer than *f2*. This new species differs from *A. lusciniae* by the following features: in females of *A. aethopygus*, the length of the stylophore is 80–90 µm; the lengths of setae *ag1* and *ag3* are 30–35 µm and 65–70 µm, respectively; fan-like setae *p’* and *p”* of legs III and IV have five or six tines; and all coxal fields are apunctate. In females of *A. lusciniae*, the length of the stylophore is 120 µm; the lengths of setae *ag1* and *ag3* are 90–115 µm and 140–155 µm, respectively; fan-like setae *p’* and *p”* of legs III and IV have seven to nine tines; and all coxal fields are punctate.

*Aulonastus arachnotherus* Sikora and Unsoeld sp. n.

(Figure 3)

Description. Female, holotype. Total body length 410 (450 in one paratype). Gnathostoma. Infracapitulum apunctate. Each medial branch of peritremes with two chambers, and each lateral branch with seven chambers. Stylophore apunctate, 90 (95) long. Idiosoma. Propodonotal shield rectangular in shape, apunctate, weakly sclerotised, bearing bases of setae *ve*, *si*, *se*, and *c1.* Setae *ve* and *si* subequal in length. Setae *c1* slightly (1.2 times) longer than *se*, and both setae situated at same transverse level. The length ratio of setae *d2:c1* 1:1.3–1.4. Hysteronotal shield fused to pygidial shield, weakly sclerotised, apunctate. Bases of setae *d1* and *e2* situated near this shield. Setae *f2* four times longer than *f1.* Setae *h2* about three times longer than *f2.* Aggenital setae *ag1* and *ag2* subequal in length, both setae shorter (about 1.5 times) than *ag3.* Genital plate absent. Both pairs of genital setae subequal in length. All coxal fields apunctate. Setae *3c* 1.5–2 times longer than *3b*. Legs. Fan-like setae *p’* and *p”* of legs III and IV with five or six tines. Lengths of setae: *ve* 20 (25), *si* 20 (25), *se* 180, *c1* 215, *c2* 180, *d1* 30, *d2* 155 (170), *e2* 30 (40), *f1* 20, *f2* 80, *h1* 20, *h2* 245, *ps1* 15, *g1* 20 (25), *g2* 20 (30), *ag1* 70 (80), *ag2* 75, *ag3* 115, *3b* 20 (30), *3c* 40 (45).

Male. Not found.

Type Material

Female holotype and one female paratype from under-tail covert quill of Long-billed Spiderhunter *Arachnothera robusta* Müller and Schlegel (Passeriformes: Nectariniidae) (host reg. no. ZSM–11.119/V169, male); Indonesia: Borneo, West Borneo Prov., Sintang, 1910, coll. L. Martin.

Type Material Deposition

Female holotype is deposited in the AMU, and female paratype is in the ZSM-SNSB.

Etymology

The name “*arachnotherus*” is derived from the generic name of the host and is formed as an adjective agreeing in gender and case with the masculine genus name *Aulonastus*. It reflects the association of this species with hosts from the genus *Arachnothera*.

Differential Diagnosis

*Aulonastus arachnotherus* sp. n. is morphologically similar to *A. nectariniiphilus* Skoracki and Zmudzinski, 2018 described from *Anthreptes reichenowi* Gunning from Tanzania [18]. In females of both species, the propodonotal shield is apunctate, the hysteronotal shield is present, and setae *f2* and *ag1* are long (about 70–110 µm). This species differs from *A. nectariniiphilus* by the following features: in females of *A. arachnotherus*, each lateral branch of the peritremes has seven chambers; the infracapitulum and coxal fields are apunctate. In females of *A. nectariniiphilus*, each lateral branch of peritremes has three to five chambers; the infracapitulum and coxal fields I and II are sparsely punctate.

*Neoaulonastus cinnyris* Klimovicova, Smolak, Njoroge and Hromada, 2014

Hosts and distribution: Uluguru Violet-backed Sunbird *Anthreptes neglectus* Neumann, Western Violet-backed Sunbird *Anthreptes longuemarei* (Lesson), both from Tanzania [18], and Eastern Double-collared Sunbird *Cinnyris mediocris* Shelley also from Tanzania [39].

*Neoaulonastus sidorchukae* Zmudzinski, Skoracki and Hromada, 2019

Host and distribution: Purple-rumped Sunbird *Leptocoma zeylonica* (Linnaeus) from Sri Lanka [19].

*Syringophiloidus haeckeli* Sikora and Unsoeld sp. n.

(Figure 4)

Description: Female, holotype. Total body length 780 (650–810 in nine paratypes). *Gnathostoma.* Infracapitulum punctate. Stylophore apunctate, 120 (110–120) long. Medial and lateral branches of peritremes with seven or eight chambers, each. Propodonotal shield apunctate, rectangular in shape, with rounded anterior margin, bearing bases of setae *vi*, *ve*, *si*, *se*, and *c1.* Setae *se* and *c1* situated at same transverse level. Length ratio of setae *vi*:*ve*:*si* 1:1.8–2:4–4.3. Hysteronotal and pygidial shields fused. Pygidial shield punctate. Setae *d2* insignificantly (1.1 times) longer than *e2*. Setae *h1* 1.3–1.5 times longer than *f1*. Setae *vi*, *ve*, *si*, *se*, *c1*, *c2*, *d1*, *d2*, and *e2* distinctly ornamented in basal part, other idiosomal setae smooth. Genital plate present, weakly sclerotised, bases of setae *ag2* and *ag3* situated on margins of this plate. Genital setae *g1* and *g2* subequal in length. Setae *ps2* 1.3–1.5 times longer than *ps1*. Length ratio of setae *ag1*:*ag2*:*ag3* 1:1–1.1:1.2. Coxal fields III–IV punctate. Setae *3c* 2–2.8 times longer than *3b*. *Legs*. Fan-like setae *p’* and *p”* of legs III and IV with five or six tines. Setae *tc”III–IV* about twice as long as *tc’III–IV*. Lengths of setae: *vi* (20–30), *ve* (40–55), *si* (85–120), *se* 200 (200–215), *c1* 210 (205–215), *c2* 195 (190–210), *d1* 170 (170–190), *d2* 180 (165–185), *e2* 150 (150–165), *f1* 15 (15–20), *f2* 210 (210–240), *h1* 20 (20–30), *h2* 305 (305–340), *ps1* 10 (10–15), *ps2* 15 (15–20), *g1* 20 (15–25), *g2* 25 (15–25), *ag1* 140 (140–155), *ag2* 150 (150–160), *ag3* 175 (170–185), *3b* 30 (25–40), *3c* 80 (70–80).

Male. Not found.

Type Material

Female holotype and nine female paratypes from quill of wing covert of Crimson Sunbird *Aethopyga siparaja* (Raffles) (Passeriformes: Nectariniidae) (host reg. no. ZSM–30.243/V168); Philippines: Luzon Island, Manila, coll. I. Marschall.

Type Material Deposition

All type specimens are deposited in the AMU, except three female paratypes are in the ZSM-SNSB.

Etymology

This species is named in honour of the German biologist, naturalist, and philosopher—Ernst Haeckel (1834–1919), well known for his notable contributions to the fields of embryology, evolution, and comparative anatomy.

Differential Diagnosis

*Syringophiloidus haeckeli* sp. n. is morphologically similar to the other species described from Sunbirds, i.e., *S. nectariniae* Skoracki and Zmudzinski, 2018. In females of both species, the infracapitulum is punctate; each lateral branch of the peritremes has seven or eight chambers; the propodonotal shield is apunctate; setae *se* and *c1* are situated at the same transverse level; and coxal fields III–IV are punctate. This new species differs from *S. nectariniae* by the following features: in females of *S. haeckeli*, the hysteronotal is fused with the pygidial shield; the pygidial shield is punctate; setae *d2* are not significantly (1.1 times) longer than *e2*; and the genital plate is present. In females of *S. nectariniae*, the hysteronotal and pygidial shields are not fused; the pygidial shield is apunctate; setae *d1* are 1.6–2 times longer than *d2*; and the genital plate is absent.

*Syringophiloidus nectariniae* Skoracki and Zmudzinski, 2018

Host and distribution: Collared Sunbird *Hedydipna collaris* (Vieillot) from Tanzania [18].

*Picobia hedydipna* Skoracki and Zmudzinski, 2018

Host and distribution: Collared Sunbird *Hedydipna collaris* (Vieillot) from Kenya [18].

*Picobia oritis* Skoracki, Antczak and Riegert, 2009

Hosts and distribution: Southern Double-collared Sunbird *Cinnyris chalybeus* (Linnaeus) from South Africa [40]; Red-chested Sunbird *Cinnyris erythrocercus* (Hartlaub) from Uganda and D.R. Congo [18,39]; Oustalet’s Sunbird *Cinnyris oustaleti* (Bocage) from Angola [39]; White-breasted Sunbird *Cinnyris talatala* Smith from Botswana and Zambia [39]; Variable Sunbird *Cinnyris venustus* (Shaw) from Somalia and Tanzania [18,39]; Olive Sunbird *Cyanomitra olivacea* (Smith) from Kenya and Tanzania [17,18]; Cameroon Sunbird *Cyanomitra oritis* (Reichenow) from Cameroon [16]; and Mouse-coloured Sunbird *Cyanomitra verreauxii* (Smith) from Tanzania [18].

### 3.2. Key to the Genera and Species of Syringophilid Mites Associated with Sunbirds (Nectariniidae)

1. Palpal tibiotarsus truncate. Tarsal setae *p’* and *p”* rod-like… subfamily Picobiinae Johnston and Kethley, 1973… 2

– Palpal tibiotarsus rounded. Tarsal setae *p’* and *p”* fan-like… subfamily Syringophilinae Lavoipierre, 1953… 3

2. Lateral propodonotal shields bear bases of setae *ve*, *si*, and *se*. Genital lobes absent. Bases of aggenital setae *ag2* situated laterally to setal bases *ag1*. Each medial branch of peritremes has three chambers… [hosts: *Cinnyris*, *Cyanomitra*]… *Picobia oritis* Skoracki, Antczak and Riegert, 2009

– Lateral propodonotal shields bear bases of setae *ve* and *si*. Genital lobes present bear genital setae. Bases of aggenital setae *ag1* and *ag2* situated in longitudinal row. Each medial branch of peritremes has seven chambers… [hosts: *Hedydipna*]… *Picobia hedydipna* Skoracki and Zmudzinski, 2018

3. Propodonotum with six pairs of setae… 4

– Propodonotum with five pairs of setae (*vi*—absent) … 8

4. Leg setae *dG* present… *Aulobia* Kethley, 1970… 5

– Leg setae *dG* absent… *Syringophiloidus* Kethley, 1970 … 7

5. Length of setae *si* 85–105 … [hosts: *Cinnyris*, *Leptocoma*]… *Aulobia nectariniae* Skoracki and Glowska, 2008

– Length of setae *si* 150–230 … 6

6. Hysteronotal shield weakly sclerotised, posterior margin does not reach bases of setae *e2* … [hosts: African species of the genus *Anthreptes*]… *Aulobia afroanthreptes* Skoracki and Zmudzinski, 2018

– Hysteronotal shield well sclerotised, posterior margin reach bases of setae *e2* … [hosts: Asian species of the genus *Anthreptes*]… *Aulobia anthreptes* Skoracki and Glowska, 2008

7. Hysteronotal and pygidial shields fused. Genital plate present… [hosts: *Aethopyga*]… *Syringophiloidus haeckeli* Sikora and Unsoeld sp. n.

– Hysteronotal and pygidial shields not fused. Genital plate absent… [hosts: *Hedydipna*]… *Syringophiloidus nectariniae* Skoracki and Zmudzinski, 2018

8. Two pairs of pseudanal setae (*ps*)… *Neoaulonastus* Skoracki, 2004… 9

– One pair of pseudanal setae present (*ps2*—absent)… *Aulonastus* Kethley, 1970… 10

9. Each medial branch of peritremes has two chambers. Lengths of setae *c2* and *d2* 90–130 and 140–160, respectively. Genital plate absent… *Neoaulonastus sidorchukae* Zmudzinski, Skoracki and Hromada, 2019

– Each medial branch of peritremes has three chambers. Lengths of setae *c2* and *d2* 155–190 and 90–115, respectively. Genital plate present… *Neoaulonastus cinnyris* Klimovicova, Smolak, Njoroge and Hromada, 2014

10. Hysteronotal shield absent. Setae *f2* and *ag1* short (30–35 µm)… [hosts: *Aethopyga*]… *Aulonastus aethopygus* Sikora and Unsoeld sp. n.

– Hysteronotal shield present. Setae *f2* and *ag1* long (70–110 µm)… 11

11. Each lateral branch of peritremes has three to five chambers. Infracapitulum and coxal fields I and II sparsely punctate… *Aulonastus nectariniiphilus* Skoracki and Zmudzinski, 2018

– Each lateral branch of peritremes has seven chambers. Infracapitulum and coxal fields apunctate… *Aulonastus arachnotherus* Sikora and Unsoeld sp. n.

### 3.3. Prevalence

To demonstrate the prevalence of infestation in Sunbird avifauna across the world, we conducted a comprehensive analysis by combining our previous research data on quill mites infesting sub-Saharan Sunbirds [18] with newly obtained data on syringophilids infesting non-African Sunbirds. Data from a total of 764 Sunbird individuals from 76 species distributed worldwide (611 individuals from 52 species in the Afrotropical region and 153 individuals from 24 non-African Sunbird species) were included in this study. Out of these, 21 host species were found to be parasitised by 12 quill mite species from five genera (Table 1). Infestation prevalences ranged from 1.4% to 100% in particular host species (Table 2). Among the analysed material, 52 species were not infested by quill mites (Table 3).

### 3.4. Host-Specificity and Bipartite Network Analysis

**Figure 5:**
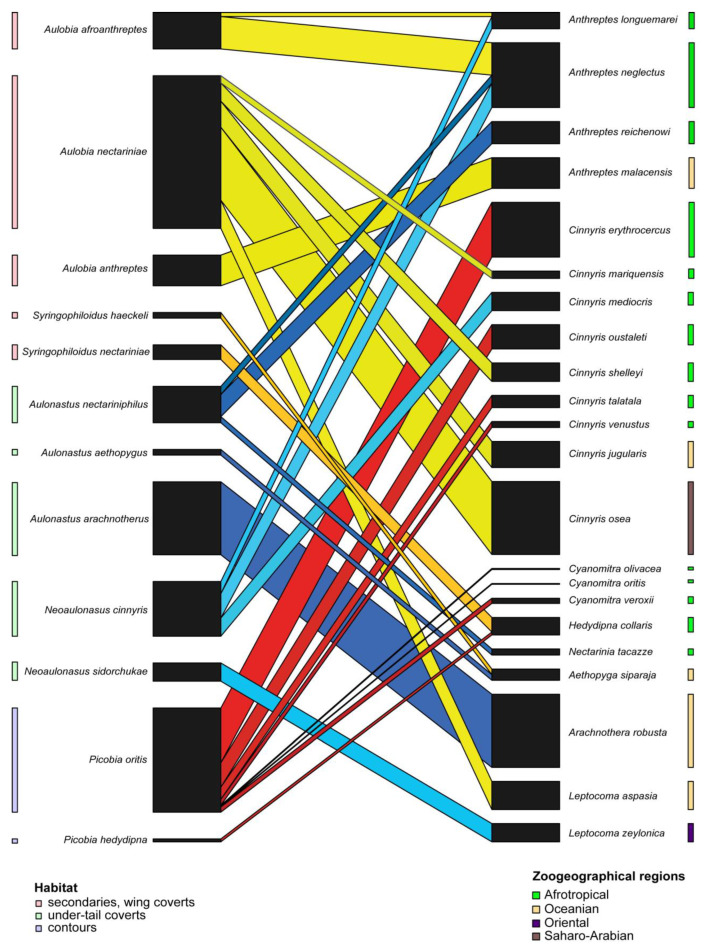
Bipartite network graph of interactions between quill mite species (**left**) and their Sunbird hosts (**right**).

**Figure 6:**
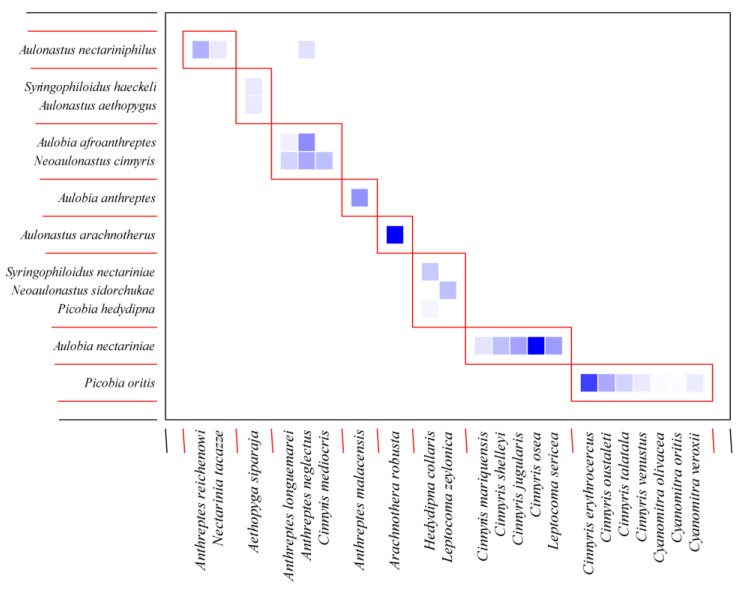
Modules (1–8), generated for quill mite species and Sunbirds. The intensity of the colours of the squares indicates the strength of the interaction between particular parasite species (vertical axis) and their host species (horizontal axis).

The Syringophilid mites–Sunbirds bipartite network (Figure 5) exhibited a low value of connectance (Con = 0.10). It means that only 10% of all possible connections between birds (hosts) and quill mites (parasites) are observed. This result is also confirmed by a C score = 0.83, indicating that syringophilids in this group of birds have a non-co-occurrence trend. Therefore, it is not surprising that the H_2′_ specialisation index = 0.94, showing that quill mites are specialised and generally do not interact with each other. A comparison between H_2′_ and null model values revealed significant differences (mean H_2′_ for null model = 0.060; t = −360.2, *p* < 0.001). The temperature of nestedness = 13.49 indicates a relatively high internal organisation of the network. The normalised specialisation level (d’) (maximum functional diversity) ranged from 0.60 to 1 (Table 4), which means that each of these parasite species has generally unique ways of interacting with the host in the network. Consequently, we observed very high modularity for our network (likelihood = 0.80), within which we identified eight modules with hosts ranging from one to seven (Figure 6).

## 4. Discussion

Quill mite fauna associated with Sunbirds. The quill mite fauna of the family Syringophilidae associated with Sunbirds currently comprises 12 species belonging to five genera and two subfamilies (Table 1). These syringophilid genera are predominantly found in birds of the order Passeriformes; in addition to Sunbirds, they are also widespread among other passerine families. For instance, the genera *Aulobia* and *Neoaulonastus* have been documented in several oscine families, while the genera *Syringophiloidus*, *Aulonastus*, and *Picobia* have been recorded on both oscine and suboscine families [14,40]. Notably, Sunbirds possess a unique parasitic fauna, where all quill mite species exclusively associate with this group of hosts as mono-, oligo-, or mesostenoxenous parasites (Table 4).

**Figure 7:**
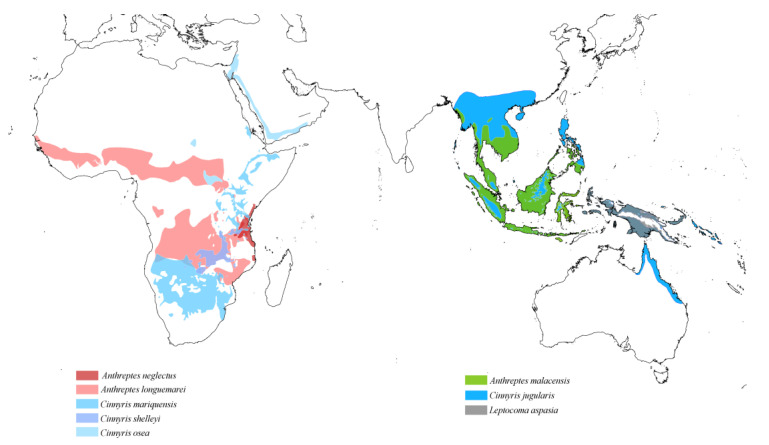
Potential distribution of quill mites of the genus *Aulobia* according to the host range.

The distribution of quill mites on Sunbirds is particularly noteworthy. Within the genus *Aulobia*, two species, *Au. afroanthreptes* and *Au. anthreptes*, are associated with birds of the genus *Anthreptes*. However, *Au. afroanthreptes* parasitises only Afrotropical Sunbirds, specifically *An. neglectus* and *An. longuemarei*, while *Au. anthreptes* is found on *An. malacensis*, which is distributed in Southeast Asia. Another species in the genus *Aulobia*, the mesostenoxenous parasite *Au. nectariniae*, has a wide host distribution. It is known from four species of the genus *Cinnyris*, which are distributed across the Afrotropical (*Ci. mariquensis* and *Ci. shelleyi*), Oceanian (*Ci. jugularis*), and Saharo-Arabian (*Ci. osea*) zoogeographical regions. Additionally, it has been recorded from *Leptocoma aspasia*, found in Eastern Indonesia and New Guinea (Figure 7).

**Figure 8:**
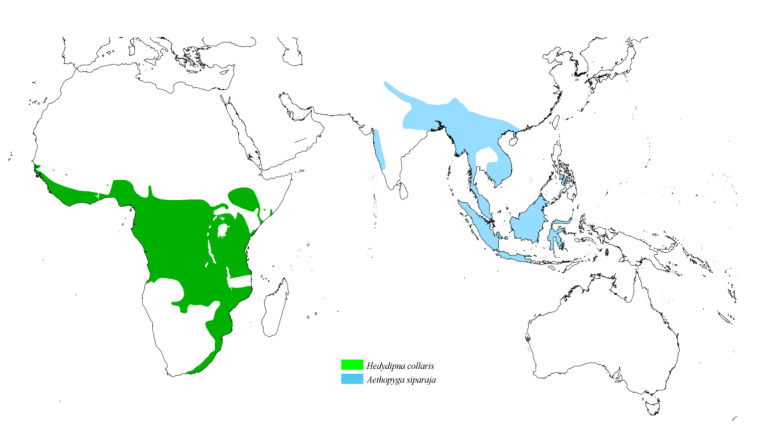
Potential distribution of quill mites of the genus *Syringophiloidus* according to the host range.

The genus *Syringophiloidus* observed on Sunbirds comprises two monoxenous species. The first, *Sy. haeckeli*, is associated with the Oceanian species *Aethopyga siparaja*, while the second, *Sy. nectariniae*, is found on the Afrotropical species *Hedydipna collaris* (Figure 8).

**Figure 9:**
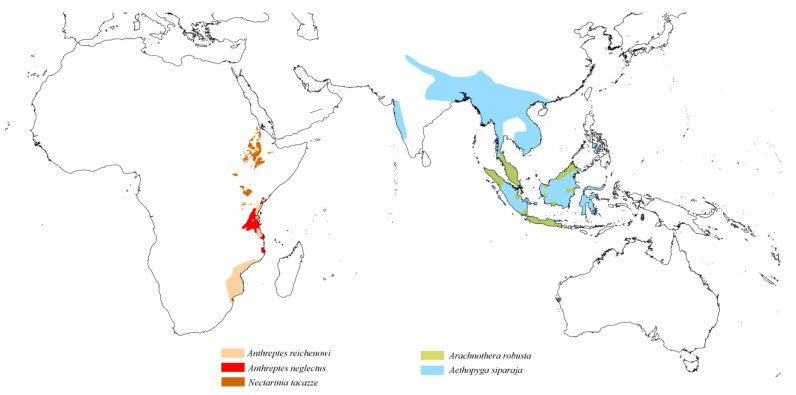
Potential distribution of quill mites of the genus *Aulonastus* according to the host range.

In the genus *Aulonastus*, two monoxenous parasites, *Aul. aethopygus* and *Aul. arachnotherus*, are associated with Oceanian Sunbirds, specifically *Aethopyga siparaja* and *Arachnothera robusta*, respectively. The third species, the mesostenoxenous parasite *Aulonastus nectariniiphilus*, is exclusively associated with Afrotropical Sunbirds belonging to the genera *Anthreptes* (*An. reichenowi* and *An. neglectus*) and *Nectarinia* (*Ne. tacazze*) (Figure 9).

**Figure 10:**
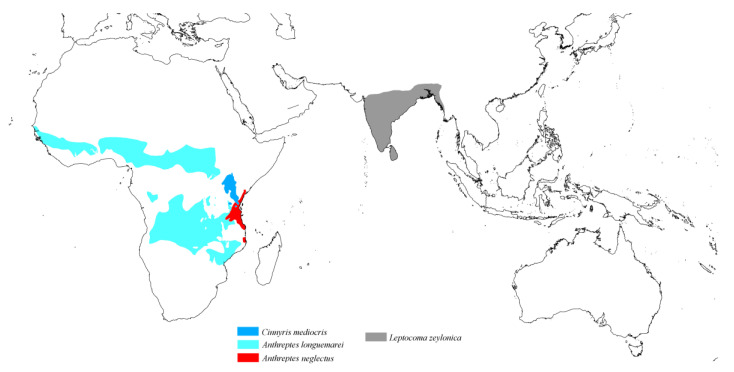
Potential distribution of quill mites of the genus *Neoaulonastus* according to the host range.

The genus *Neoaulonastus*, associated with Sunbirds, includes two mite species. The monoxenous species *N. sidorchukae* occupies the Oriental Sunbird species *Leptocoma zeylonica*, while *N. cinnyris* parasitises Afrotropical Sunbirds of the genera *Anthreptes* (*A. neglectus* and *A. longuemarei*) and *Cinnyris* (*C. mediocris*) (Figure 10).

**Figure 11:**
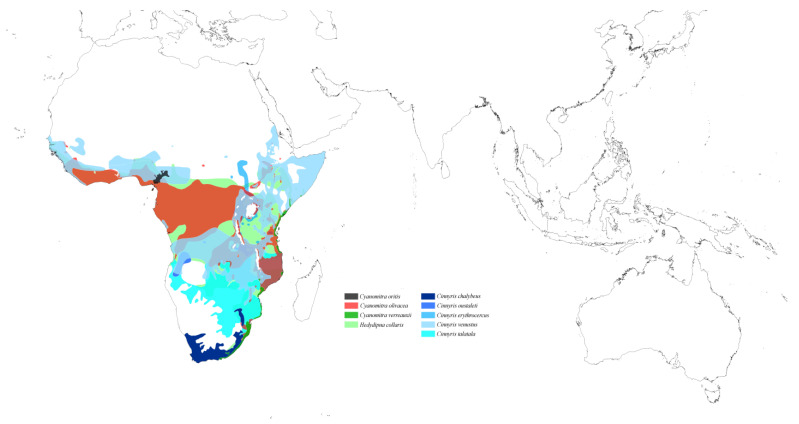
Potential distribution of quill mites of the genus *Picobia* according to the host range.

The last genus, *Picobia*, is the only one from the subfamily Picobiinae and is represented by two species of Sunbirds. The first species, the monoxenous *Pi. hedydipna*, is associated with the Afrotropical host species *Hedydipna collaris*. The second species, *Pi. oritis*, belongs to the group of mesostenoxenous parasites inhabiting a broad spectrum of Afrotropical Sunbirds. Hosts of this parasite include representatives of the genera *Cinnyris* (*Ci. chalybeus*, *Ci. erythrocercus*, *Ci. oustaleti*, *Ci. talatala*, and *Ci. venustus*) and *Cyanomitra* (*Cy. olivacea*, *Cy. oritis*, and *Cy. verreauxii*) (Figure 11).

Based on the above data, the mite parasites associated with Sunbirds can be divided into three groups: Group 1: Quill mites restricted to the Afrotropical Sunbirds: *Aulobia afroanthreptes*, *Syringophiloidus nectariniae*, *Aulonastus nectariniiphilus*, *Picobia hedydipna*, and *Picobia oritis*; Group 2: Quill mites restricted to the non-Afrotropical Sunbirds: *Aulobia anthreptes*, *Syringophiloidus haeckeli*, *Aulonastus aethopygus*, and *Aulonastus arachnotherus*; Group 3: Quill mites parasitising both Afrotropical and non-Afrotropical Sunbirds: only *Aulobia nectariniae*.

The origin of Sunbirds can be traced back to the Old World, with their evolutionary roots believed to be in Africa. Sunbirds likely evolved in Africa and subsequently dispersed to other regions, including Asia and the Pacific islands. This suggests that before Sunbird diversification, the quill mite fauna has been represented by all four quill mite genera, (*Aulobia*, *Aulonastus*, *Syringophiloidus*, and *Picobia*) present on Afrotropical Sunbirds (Group 1). The absence of *Picobia* representatives on Asian Sunbirds suggests that these mites may have been lost during the birds’ evolutionary dispersion from Africa to Asia.

Habitat specificity. The two genera belonging to the subfamily Syringophilinae, *Aulobia* and *Syringophiloidus*, consist of large-sized syringophilids inhabiting quills of secondary feathers and wing coverts. The other two syringophiline genera, *Aulonastus* and *Neoulonastus*, include small-sized syringophilids that were found in the small quills of under-tail coverts. The last genus, *Picobia*, the only one from the subfamily Picobiinae, is exclusively found in the quills of contour feathers and is represented by two species.

Prevalence and bipartite network of the quill mites and Sunbirds communities. Research on the infestation of wild birds by the syringophilid mites has started relatively recently. The prevalence (IP—index of prevalence) of infestation by quill mites is relatively low, particularly among wild, non-social, and independently breeding hosts. This suggests that mites that disperse through means other than nestling passage or host copulation have very limited opportunities to colonise new hosts. However, in gregarious hosts, the IP is higher, with the highest values observed in social and domestic birds [41,42,43,44,45,46,47,48]. Based on the results of this study, host species can be grouped into four classes according to the prevalence index: low IP 1–25% (*Anthreptes longuemarei*, *An. malacensis*, *Aethopyga siparaja*, *Cinnyris mariquensis*, *Ci. mediocris*, *Ci. shelleyi*, *Ci. talatala*, *Ci. venustus*, *Cyanomitra olivacea*, *Cy. veroxii*, *Hedydipna collaris*, *Leptocoma zeylonica*, and *Nectarinia tacazze*); middle IP 26–50% (*Anthreptes reichenowi*, *Cinnyris jugularis*, *Ci. oustaleti*, and *Leptocoma aspasia*); high IP 51–75% (*Cinnyris erythrocercus*); and extremely high 76–100% (*Anthreptes neglectus*, *Arachnothera robusta*, and *Cinnyris osea*).

The bipartite network analysis of the interactions between Sunbirds and their parasitic quill mites has revealed intricate ecological relationships, marked by a high degree of specialisation (H_2′_ = 0.94). This indicates that most quill mite species are adapted to infest specific Sunbird hosts, suggesting co-evolutionary dynamics driven by morphological, behavioural, or ecological adaptations. The prevalence of infestation varies significantly across different bird species, ranging from 1.4% to 100%, which may be attributed to variations in bird behaviour, habitat, and immunity to infestation. The network’s high modularity (likelihood = 0.80) reveals distinct clusters of interactions within the community, indicating stronger interactions within certain host–parasite groups and weaker connections between them. The variation in the degree of specialisation among individual parasite species, represented by the d’ index, suggests a spectrum of parasite–host relationship dynamics. Understanding these patterns is crucial for biodiversity conservation efforts, as it sheds light on potential threats to bird populations from parasitic infestations and helps in formulating targeted conservation strategies. Additionally, these findings highlight the significance of ongoing research into host–parasite relationships and their impact on ecosystem dynamics, including how environmental changes influence these interactions and how birds’ defence mechanisms against parasites evolve. In summary, this study not only provides valuable insights into the complex host–parasite interactions within ecosystems but also highlights the significance of these interactions in understanding biodiversity and ecosystem conservation.

## 5. Conclusions

This study highlights the remarkable diversity and specialisation of quill mites (Syringophilidae) associated with Sunbirds (Nectariniidae), underscoring the intricate ecological relationships between these parasites and their avian hosts. The findings reveal that Sunbirds harbour a unique parasitic fauna, with quill mites demonstrating varying degrees of host specificity, ranging from monoxenous to mesostenoxenous associations. The geographic distribution of these mites further reflects the evolutionary history of Sunbirds, suggesting that certain mite genera may have been lost during the birds’ dispersion from Africa to Asia. The study also emphasises the habitat specificity of quill mites, with different genera occupying distinct regions of the feathers, from wing coverts to contour feathers, showcasing their adaptation to specific microhabitats on their hosts. Furthermore, the prevalence and bipartite network analyses indicate a high degree of specialisation and modularity within the Sunbird–quill mite communities, suggesting co-evolutionary dynamics driven by host behaviour. These findings contribute valuable insights into the complex host–parasite interactions within ecosystems and highlight the importance of continued research in this area. Understanding these dynamics is crucial for biodiversity conservation, as it sheds light on potential threats to bird populations from parasitic infestations and informs targeted conservation strategies. The study underscores the significance of host–parasite relationships in maintaining ecosystem health and biodiversity, particularly in the face of environmental changes.

## Figures and Tables

**Figure 2:**
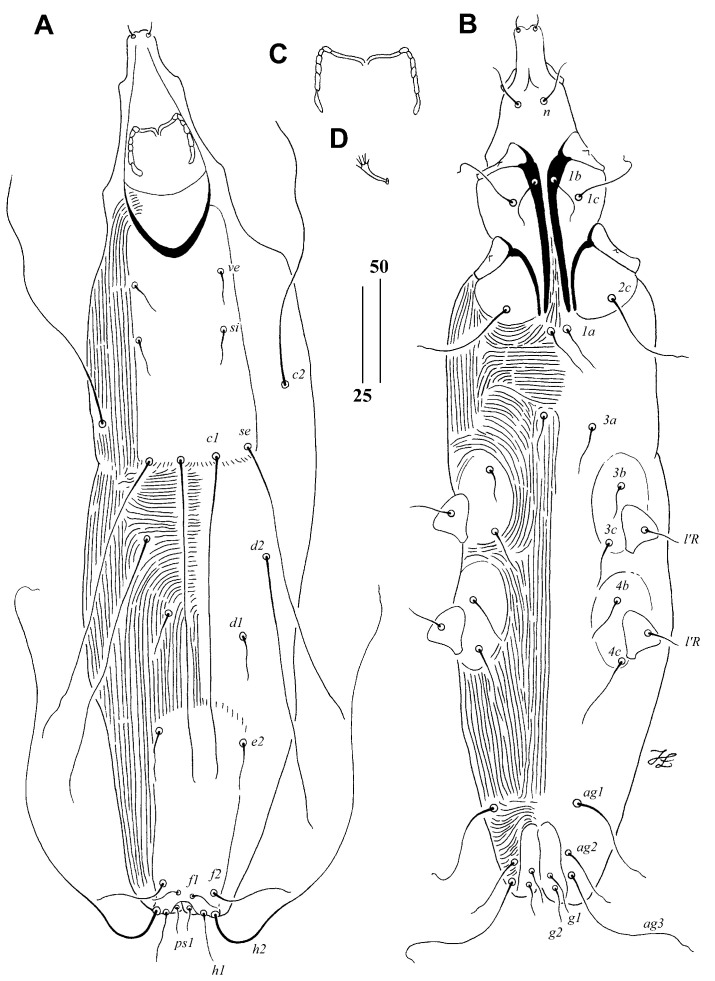
*Aulonastus aethopygus* Sikora and Unsoeld sp. n., female. (**A**)—dorsal view; (**B**)—ventral view; (**C**)—peritremes; (**D**)—fan-like seta *p’III*. Scale bars: (**A**,**B**) = 50 µm, (**C**,**D**) = 25 µm.

**Figure 3:**
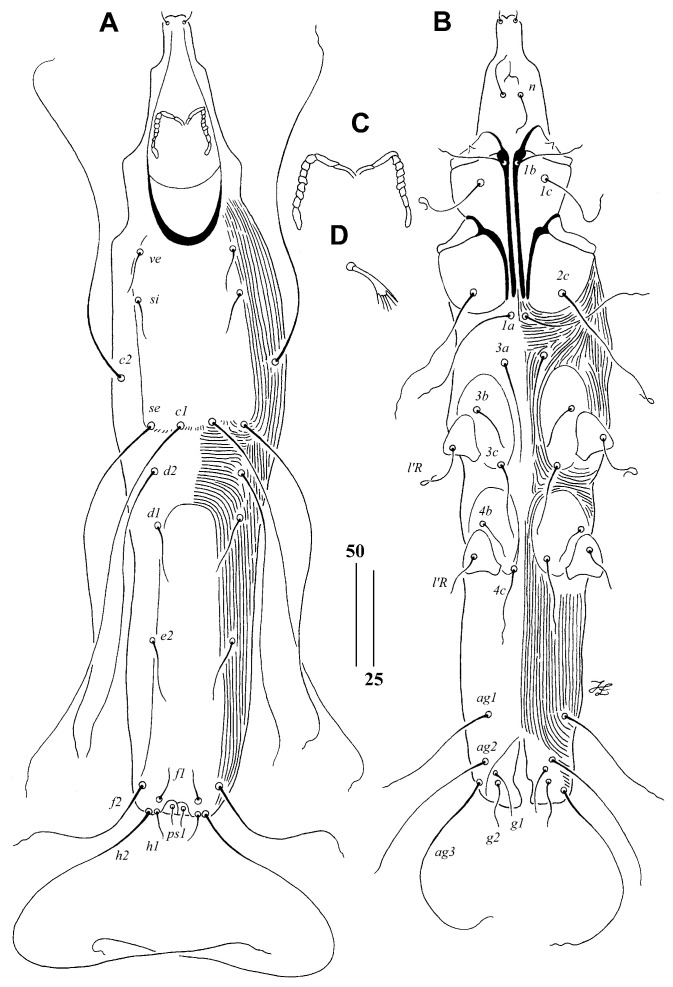
*Aulonastus arachnotherus* Sikora and Unsoeld sp. n., female. (**A**)—dorsal view; (**B**)—ventral view; (**C**)—peritremes; (**D**)—fan-like seta *p’III*. Scale bars: (**A**,**B**) = 50 µm, (**C**,**D**) = 25 µm.

**Figure 4:**
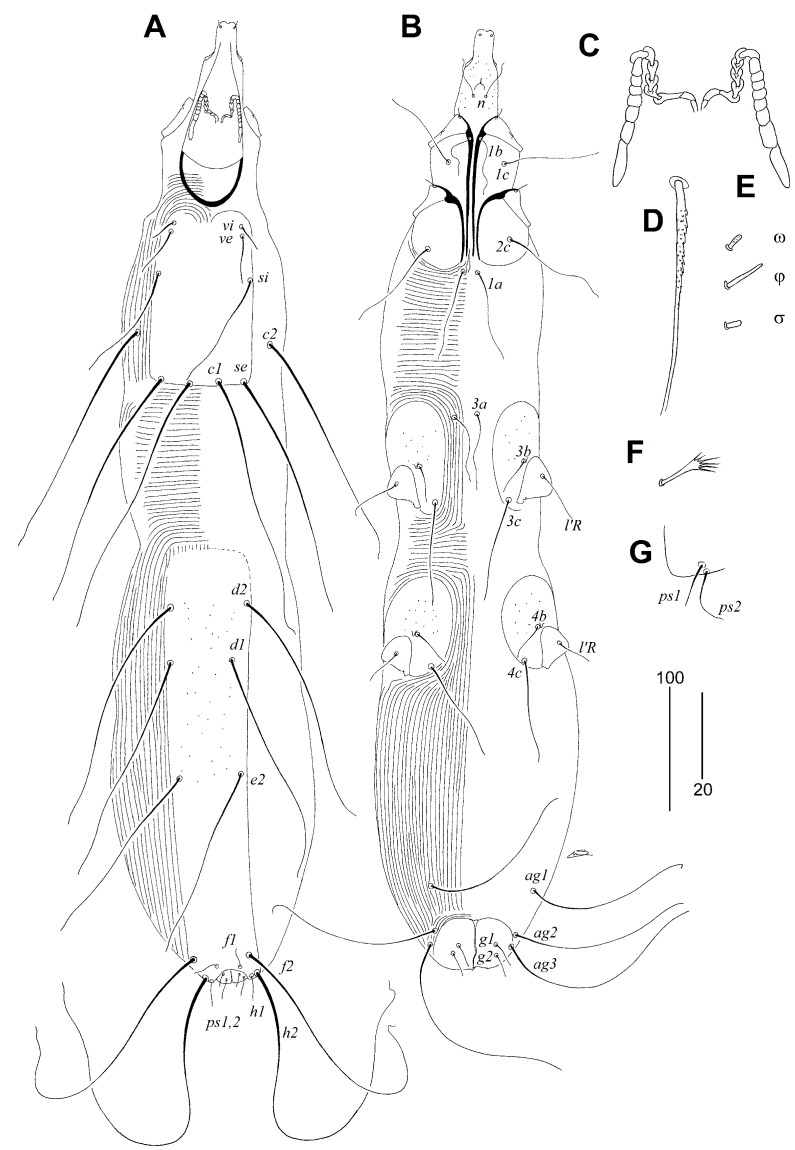
*Syringophiloidus haeckeli* Sikora and Unsoeld sp. n., female. (**A**)—dorsal view; (**B**)—ventral view; (**C**)—peritremes; (**D**)—propodonotal seta *si*; (**E**)—solenidia of leg I; (**F**)—fan-like seta *p’III*; (**G**)—pseudanal setae *ps1–2*. Scale bars: (**A**,**B**) = 50 µm, (**C**–**G**) = 25 µm.

**Table 1:** Quill mites of the family Syringophilidae parasitising Sunbirds (Nectariniidae). *—type host species; sec—secondary; w-cov—wing covert; utc—under-tail covert; con—contour feathers; zoogeographical regions: Afro—Afrotropical; Ocea—Oceanian; Orie—Oriental; Saha-Arab—Saharo-Arabian.

Quill Mite Species	Host	Habitat	Distribution	References
*Aulobia afroanthreptes* Skoracki and Zmudzinski, 2018	*Anthreptes neglectus* Neumann *	sec, w-cov	Afro: Tanzania	[18]
“	*Anthreptes longuemarei* (Lesson)	sec, w-cov	Afro: Tanzania	[18]
*Aulobia anthreptes* Skoracki and Glowska, 2008	*Anthreptes malacensis* (Scopoli)	sec, w-cov	Ocea: SE Asia; Indonesia (Java)	[15]; current study
*Aulobia nectariniae* Skoracki and Glowska, 2008	*Cinnyris jugularis* (Linnaeus) *	sec, w-cov	Ocea: Papua New Guinea	[15]; current study
“	*Cinnyris mariquensis* Smith	sec, w-cov	Afro: Tanzania	[18]
“	*Cinnyris shelleyi* Alexander	sec, w-cov	Afro: Tanzania	[18]
“	*Cinnyris osea* Bonaparte	w-cov	Saha-Arab: Palestine	current study
“	*Leptocoma aspasia* (Lesson and Garnot)	sec, w-cov	Ocea: Papua New Guinea	[15]; current study
*Aulonastus nectariniiphilus* Skoracki and Zmudzinski, 2018	*Anthreptes reichenowi* Gunning *	utc	Afro: Tanzania	[18]
“	*Nectarinia tacazze* (Stanley)	utc	Afro: Ethiopia	[18]
*Aulonastus aethopygus* Sikora and Unsoeld sp. n.	*Aethopyga siparaja* (Raffles)	utc	Ocea: Philippines	current study
*Aulonastus arachnotherus* Sikora and Unsoeld sp. n.	*Arachnothera robusta* Müller and Schlegel	utc	Ocea: Indonesia (Borneo)	current study
*Neoaulonastus sidorchukae* Zmudzinski, Skoracki and Hromada, 2019	*Leptocoma zeylonica* (Linnaeus)	utc	Orie: Sri Lanka	[19]
*Neoaulonastus cinnyris* Klimovicova, Smolak, Njoroge and Hromada, 2014	*Anthreptes neglectus* Neumann	utc	Afro: Tanzania	[18]
“	*Athreptes longuemarei* (Lesson)	utc	Afro: Tanzania	[18]
“	*Cinnyris mediocris* Shelley *	utc	Afro: Tanzania	[39]
*Syringophiloidus haeckeli* Sikora and Unsoeld sp. n.	*Aethopyga siparaja* (Raffles)	w-cov	Ocea: Philippines	current study
*Syringophiloidus nectariniae* Skoracki and Zmudzinski, 2018	*Hedydipna collaris* (Vieillot)	w-cov	Afro: Tanzania	[18]
*Picobia hedydipna* Skoracki and Zmudzinski, 2018	*Hedydipna collaris* (Vieillot)	con	Afro: Kenya	[18]
*Picobia oritis* Skoracki, Antczak and Riegert, 2009	*Cinnyris chalybeus* (Linnaeus)	con	Afro: South Africa	[18]
“	*Cinnyris erythrocercus* (Hartlaub)	con	Afro: Uganda, D.R. Congo	[18,39]
“	*Cinnyris oustaleti* (Bocage)	con	Afro: Angola	[39]
“	*Cinnyris talatala* Smith	con	Afro: Botswana, Zambia	[39]
“	*Cinnyris venustus* (Shaw)	con	Afro: Somalia, Tanzania	[18,39]
“	*Cyanomitra olivacea* (Smith)	con	Afro: Kenya, Tanzania	[17,18]
“	*Cyanomitra oritis* (Reichenow) *	con	Afro: Cameroon	[16]
“	*Cyanomitra verreauxii* (Smith)	con	Afro: Tanzania	[18]

**Table 2:** Sunbirds and their quill mite parasites (compilation of current study (indicated by asterisk) and Skoracki et al. [18]).

Host Species	Examined/Infested	Prevalence (CI^Sterne method^)	Quill Mite Species
*Anthreptes longuemarei*	18/3	16.7 (4.7–41.4)	*Neoaulonastus cinnyris*
”	18/1	5.6 (0.3–27.1)	*Aulobia afroanthreptes*
*Anthreptes malacensis* *	24/1	42 (0.2–20.4)	*Aulobia anthreptes*
*Anthreptes neglectus*	9/4	44.4 (16.9–74.9)	*Aulobia afroanthreptes*
”	9/3	33.3 (9.8–67.7)	*Neoaulonastus cinnyris*
”	9/1	11.1 (0.6–44.4)	*Aulobia afroanthreptes + N. cinnyris*
”	9/1	11.1 (0.6–44.4)	*Aulonastus nectariniphilus*
*Anthreptes reichenowi*	23/7	30.4 (14.5–52.2)	*Aulonastus nectariniphilus*
*Aethopyga siparaja* *	13/1	7.7 (0.4–34.2)	*Aulonastus aethopygus + Syringophiloidus haeckeli*
*Arachnothera robusta* *	1/1	100 (50–100)	*Aulonastus arachnotherus*
*Cinnyris erythrocercus*	4/3	75.0 (24.9–98.7)	*Picobia oritis*
*Cinnyris jugularis* *	14/5	35.7 (15.3–62.9)	*Aulobia nectariniae*
*Cinnyris osea* *	1/1	100 (50–100)	*Aulobia nectariniae*
*Cinnyris mariquensis*	20/2	10.0 (1.8–32.0)	*Aulobia nectariniae*
*Cinnyris mediocris*	4/1	25.0 (1.3–75.1)	*Neoaulonastus cinnyris*
*Cinnyris oustaleti*	3/1	33.3 (1.7–86.5)	*Picobia oritis*
*Cinnyris shelleyi*	12/3	25.0 (7.2–54.3)	*Aulobia nectariniae*
*Cinnyris talatala*	12/2	16.7 (3.0–45.7)	*Picobia oritis*
*Cinnyris venustus*	37/3	8.1 (2.2–21.4)	*Picobia oritis*
*Cyanomitra olivacea*	73/1	1.4 (0.1–7.3)	*Picobia oritis*
*Cyanomitra veroxii*	14/1	7.1 (0.4–31.7)	*Picobia oritis*
*Hedydipna collaris*	25/5	20.0 (8.2–39.8)	*Syringophiloidus nectariniae*
”	25/1	4.0 (0.2–19.6)	*Picobia hedydipna*
*Leptocoma aspasia* *	13/5	38.6 (16.6– 65.8)	*Aulobia nectariniae*
*Leptocoma zeylonica* *	8/2	25 (4.6–63.5)	*Neoaulonastus sidorchukae*
*Nectarinia tacazze*	12/1	8.3 (0.4–37.0)	*Aulonastus nectariniphilus*

Cyanomitra oritis N/A.

**Table 3:** Examined Sunbirds uninfested by syringophilid mites (compilation of current study (indicated by asterisk) and Skoracki et al. [18]).

Sunbird Species	Examined	Sunbird Species	Examined
*Aethopyga christinae* *	8	*Cinnyris chloropygius*	1
*Aethopyga eximia* *	1	*Cinnyris cupreus* *	40
*Aethopyga ignicauda* *	5	*Cinnyris habessinicus*	4
*Aethopyga mystacalis* *	5	*Cinnyris johannae*	1
*Aethopyga nipalensis* *	11	*Cinnyris lotenius* *	3
*Aethopyga saturata* *	5	*Cinnyris loveridgei*	8
*Anthreptes orientalis*	1	*Cinnyris minullus* *	7
*Anthreptes rectirostris*	2	*Cinnyris moreaui* *	4
*Anthreptes rubritorques*	4	*Cinnyris nectarinoides*	1
*Anthreptes seimundi*	5	*Cinnyris notatus*	1
*Arachnothera affinis* *	8	*Cinnyris pembae* *	17
*Arachnothera chrysogenys* *	3	*Cinnyris pulchellus*	1
*Arachnothera flavigaster* *	1	*Cinnyris regius*	1
*Arachnothera longirostra* *	7	*Cinnyris reichenowi*	2
*Arachnothera magna* *	1	*Cinnyris solaris* *	2
*Chalcomitra amethystina*	48	*Cinnyris superba*	1
*Chalcomitra fuliginosa*	1	*Cyanomitra alinae* *	12
*Chalcomitra hunteri*	12	*Cyanomitra cyanolaema* *	7
*Chalcomitra rubescens*	10	*Cyanomitra verticalis*	10
*Chalcomitra senegalensis*	15	*Drepanorhynchus reichenowi* *	3
*Chalcoparia singalensis*	1	Leptocoma calcostetha*	2
*Cinnyris afer*	2	*Leptocoma sperata* *	5
*Cinnyris asiaticus* *	9	*Nectarinia famosa*	1
*Cinnyris bifasciatus*	13	*Nectarinia johnstoni* *	75
*Cinnyris bouvieri* *	8	*Nectarinia kilimensis*	1
*Cinnyris chalybeus* *	16	*Nectarinia purpureiventris*	2

**Table 4:** Host specificity of quill mite species with the value of d’ index.

Quill Mite Species	d’	Mite Species	Host Spectrum
Monoxenous parasites	1	*Aulobia anthreptes*	*Anthreptes malacensis*
	0.82	*Aulonastus aethopygus*	*Aethopyga siparaja*
	1	*Aulonastus arachnotherus*	*Arachnothera robusta*
	1	*Neoaulonastus sidorchukae*	*Leptocoma zeylonica*
	0.82	*Syringophiloidus haeckeli*	*Aethopyga siparaja*
	0.94	*Syringophiloidus nectariniae*	*Hedydipna collaris*
	0.6	*Picobia hedydipna*	*Hedydipna collaris*
Oligoxenous parasites	0.71	*Aulobia afroanthreptes*	*Anthreptes neglectus*
			*Anthreptes longuemarei*
Mesostenoxenous parasites	1	*Aulobia nectariniae*	*Cinnyris jugularis*
			*Cinnyris mariquensis*
			*Cinnyris shelleyi*
			*Cinnyris osea*
			*Leptocoma aspasia*
	0.82	*Aulonastus nectariniiphilus*	*Anthreptes reichenowi*
			*Nectarinia tacazze*
	0.77	*Neoaulonastus cinnyris*	*Anthreptes neglectus*
			*Athreptes longuemarei*
			*Cinnyris mediocris*
	1	*Picobia oritis*	*Cinnyris chalybeus*
			*Cinnyris erythrocercus*
			*Cinnyris oustaleti*
			*Cinnyris talatala*
			*Cinnyris venustus*
			*Cyanomitra olivacea*
			*Cyanomitra oritis*
			*Cyanomitra verreauxii*

## Data Availability

All necessary data are available in the text for this article.
